# The influence of perceived design source (AI vs. human) on Chinese consumers’ purchase intention: The mediating roles of novelty, perceived brand effort, and product type

**DOI:** 10.1371/journal.pone.0346737

**Published:** 2026-04-16

**Authors:** Yunxi Feng, Yibei Ran

**Affiliations:** 1 School of Art and Design, Zhengzhou University of Aeronautics, Zhengzhou, China; 2 International Fine Arts, Kyonggi University, Suwon, South Korea; Gannon University, UNITED STATES OF AMERICA

## Abstract

Product design is a crucial source of competitive advantage for companies and greatly influences their market position. Information about “who designed the product,” known as the perceived design source, is gaining widespread attention in product design communication. Due to limitations in existing research, this study, based on 316 valid questionnaires, used Mplus 8.0 to examine how the perceived design source (AI versus human) affects Chinese consumers’ purchase intentions. The research findings are as follows: (1) Consumers are more willing to buy products perceived as designed by AI compared to those designed by humans; (2) Novelty mediates the relationship between the perceived design source and consumers’ purchase intentions; (3) Perceived brand effort also mediates this relationship; (4) Product type moderates the relationship between the perceived design source and purchase intentions. These conclusions not only advance marketing theory but also help businesses develop targeted marketing strategies and increase consumer purchasing.

## 1. Introduction

Product design is a vital source of competitive advantage for companies and greatly affects their market positions [1,2]. Information about “who designed the product,” namely the perceived design source, is increasingly a key element in product communication and consumer assessment [3–5]. Historically, professional human designers have dominated this area [6]. However, the ongoing development of AI technology is fundamentally changing this field. AI’s ability to accurately interpret external data, learn from it, and adapt to meet goals [7] has allowed its entry into areas once thought to rely solely on human creativity [8–10]. Real examples of this trend already exist. For instance, in 2018, an AI-created portrait sold at Christie’s for $432,500, demonstrating AI’s potential in art creation. In 2019, an AI-designed garment won a top award at a major international fashion contest, further demonstrating its practical capabilities. These developments highlight that integrating AI into product design is not merely a future prospect but a current reality, underscoring the importance of understanding how consumers respond to this new design approach compared with traditional human designers.

Research on how perceived design source information affects consumer preferences reveals a complex and often conflicting picture. While prior studies have begun comparing different sources, findings are fragmented and conflicting: some studies report a positive effect of AI design [5,11–13], whereas others report a negative effect [4,10,11,14–17]. In fact, merely noting the existence of this contradiction or the need for direct AI-human comparison is insufficient. In other words, existing research has a more fundamental theoretical gap: the absence of an integrated framework that can simultaneously account for the opposing effects (positive vs. negative) of AI design on consumer evaluation reported in the literature. The first key issue this study aims to address is the proposal and testing of a simple, comprehensive theoretical system that accounts for these divergent outcomes. Additionally, past research has often examined isolated mediators or moderators across different product contexts [4,18–20], but has not modeled the competing psychological pathways that function simultaneously. This is the second key issue this study seeks to resolve.

This study is designed to address these two core issues: the introduction and empirical validation of a dual-pathway cognitive evaluation framework grounded in Cognitive Evaluation Theory. This framework suggests that consumers evaluate AI versus human-designed sources through two parallel yet opposing cognitive appraisals: a novelty-driven positive pathway and a perceived-effort-based negative pathway. The construct of novelty captures the positive appraisal in which AI’s non-human origin creates schema incongruity [21], triggering arousal and perceptions of innovative value [22,23]. In contrast, perceived brand effort captures the negative appraisal, in which AI’s automated nature may be heuristically associated with lower emotional commitment and labor investment [12,24,25], thereby reducing perceived value. By integrating these pathways, our model directly explains the central contradiction in the literature as the net outcome of competing forces rather than as context-bound anomalies, thereby synthesizing insights from schema- and effort-based perspectives [16,17]. Additionally, we introduce product type (utilitarian vs. hedonic) not just as another moderator but as the critical boundary condition that activates one primary pathway over the other, thereby reconciling inconsistent findings across product categories [16,17,26]. Therefore, this study’s uniqueness lies in providing a cohesive, testable model that systematically explains when and why perceived AI design yields higher or lower purchase intentions than perceived human design, integrating diverse prior findings into a unified theoretical narrative.

## 2. Theoretical framework and hypothesis development

### 2.1. Theoretical framework

This study uses Cognitive Evaluation Theory (CET) as its primary framework to connect key concepts and explain their relationships [27,28]. CET shows that individuals’ emotional and behavioral responses are not immediate reactions to external events but are shaped by their mental assessment of how those events affect their well-being [29]. This stimulus-appraisal-outcome process supports the core idea in consumer psychology, where external and internal stimuli lead to evaluative and behavioral results through cognitive and emotional processing [30,31]. We adapt this model to fit consumer contexts, treating the perceived design source (AI versus human) as a key external stimulus that initiates a cognitive evaluation, which in turn influences the primary behavioral outcome: purchase intention [32,33].

Within this framework, the perceived design source serves as the primary external stimulus for assessment [12]. When consumers encounter this stimulus, they are hypothesized to engage in a multidimensional appraisal that unfolds along two main axes, each reflecting distinct value judgments. First, novelty serves as the dimension of innovative potential and schema incongruity. It assesses the stimulus as new, original, and unexpected [34]. This appraisal can be positive, encouraging goal pursuit and signaling future-oriented value [35], and is recognized as a key cognitive pathway that translates various stimuli into purchase intentions [36,37]. Second, perceived brand effort functions as the dimension of invested authenticity and value. It involves evaluating the intentionality, emotional labor, and craftsmanship inferred from the perceived design source [38]. This appraisal is crucial in creative and experiential contexts, where perceiving greater effort is associated with heightened authenticity and intrinsic value—a pattern observed across settings ranging from cultural products to digital experiences [37,39].

Critically, the appraisal process is not uniform; it is inherently context-dependent. Product type (utilitarian vs. hedonic) establishes the fundamental appraisal context that determines the personal relevance, salience, and weighting of the two appraisal dimensions [40]. This moderating role is well documented: product type systematically directs whether cognitive attributes such as novelty and usefulness, or affective attributes related to authenticity and effort, are prioritized in consumer judgment [41,42], thereby influencing which pathway dominates the decision-making process [43,44]. For a utilitarian product, where the main goal is functional problem-solving, the novelty dimension becomes more prominent. Conversely, for a hedonic product, where the goal is experiential and emotional fulfillment, the effort dimension is emphasized. Thus, product type systematically guides the focus of the cognitive appraisal. The overall outcome of this contextualized appraisal process is the formation of purchase intention [45]. By framing all variables within this integrated CET framework, we offer a concise yet robust rationale for how and why the perceived design source influences purchase decisions via distinct, context-dependent cognitive pathways.

### 2.2. AI usage and consumers’ purchase intention

Although existing research has explored the correlation between perceived AI design and consumers’ purchase intention, the literature presents conflicting findings: several studies report a positive effect of perceived AI design on consumer preference [5,11–13], while others report a negative effect [4,10,11,14,15]. This lack of consensus, combined with the specific population and context of the present study, necessitates a verification of this fundamental relationship. Relevant research shows that the cultural meanings carried by celebrities and symbols can be transferred to associated products and brands, thereby boosting brand value and market performance. [46–48]. The product’s design serves as a “silent spokesperson.” When a brand clearly indicates that a product is designed by AI, the positive attributes of AI technology, such as “cutting-edge technology,” “modernity,” and “innovation,” will be transferred to the product, thereby enhancing its appeal and brand image [5]. When consumers choose products, they are often influenced by the cultural meanings attached to the products.

From a cognitive mechanisms perspective, perceived AI-designed products can elicit higher levels of perceived novelty among consumers, thereby promoting purchase intention. Research shows that when a product design significantly differs from consumers’ existing mental models, it triggers stronger cognitive conflicts and prompts more exploration [21]. The AI-generated design solutions are unique and unconventional, which readily elicits consumers’ perceptions of novelty. This sense of novelty, through emotional arousal and interest stimulation, enables consumers to form a more positive evaluation of the product [49]. Especially in the field of utilitarian products, the technical-rational characteristics of perceived AI-designed products are highly consistent with functional requirements, thereby enhancing consumer acceptance [12].

In practical terms, the practical advantages of AI in design strengthen consumers’ positive responses. In the creative generation stage, AI can process massive amounts of data, identify potential trends, and generate innovative solutions that human designers may overlook [50,51]. This means that AI-designed products may have a broader creative scope and a forward-looking orientation, thereby meeting consumers’ ever-changing needs. In the solution optimization stage, AI can integrate multiple sources of information and mitigate cognitive biases in human decision-making [52], thereby making product design more scientific and rational. In addition, AI can efficiently perform repetitive tasks, reduce design costs, and enable enterprises to allocate more resources to innovation and breakthroughs [7]. These technological advantages enable consumers to associate AI-designed products with attributes such as “more scientific,” “more efficient,” and “more accurate,” thereby enhancing their perception of product value. When consumers purchase products, they seek products with high cost-effectiveness, high quality, and innovation. The advantages of AI-designed products in these aspects align with consumers’ expectations, thereby increasing their willingness to purchase [53]. Based on the preceding analysis, the following hypothesis is proposed to establish the foundational applicability of our theoretical model within the specific research context:

H1: Consumers are more willing to buy products perceived as designed by AI compared to those designed by humans.

### 2.3. The mediating role of novelty

Changes in product characteristics or related factors can trigger consumers’ perception of novelty [54,55]. When AI technology, which represents intelligence and automation, is introduced into products, it creates a significant cognitive contrast. The application of AI technology deviates from consumers’ existing cognitive models of products, constituting cognitive novelty. Research shows that when a stimulus doesn’t match an individual’s existing mental categories, it is perceived as novel [21]. Therefore, the introduction of AI into product design can significantly enhance consumers’ perceptions of novelty. Additionally, new technologies or production methods, such as augmented reality and upcycling, have been shown to enhance novelty [56,57]. AI provides consumers with a brand-new experience by transforming traditional product development models (e.g., AI-generated design, algorithm-driven creativity), further enhancing its novelty attribute [4,9]. The “black-box” nature of AI and the opacity of its decision-making process also reinforce the common-sense association of AI with novelty [58,59].

As an emotional belief, novelty is an important determinant of attitude formation [49]. Consumers often regard the novelty of new products as the added value and attribute optimization provided by enterprises [60]. It can not only enhance emotional arousal, stimulate interest and positive emotions [22], but also contribute to the formation of a positive product attitude [23]. From a visual packaging perspective, a novel design can enhance product attractiveness, influence consumers’ evaluations of quality and satisfaction, and thereby shape purchase intentions [61,62]. Unique packaging helps products stand out, enhances consumer interaction, and meets consumers’ psychological need for uniqueness. Consumers use innovative products to enhance their personal and social identity, and their perception of innovation indirectly promotes purchase intention through factors such as trust [63], emotional satisfaction [64], and product attitude [65]. Therefore, a higher perception of novelty not only strengthens consumers’ recognition of product differentiation and social value [66,67] but also significantly increases their purchase intention. Based on the above analysis, this study proposes the following hypothesis:

H2: Novelty mediates the relationship between perceived design source and consumers’ purchase intention.

### 2.4. The mediating role of perceived brand effort

Research confirms that the price of a commodity reflects its intrinsic value, and the magnitude of that value is determined by socially necessary labor time [25]. Since AI-based design requires less labor time, it is considered to have lower value; accordingly, the price should also be lower. Although AI technology can improve efficiency, reduce costs, and achieve higher precision [12], with the remarkable advances in generative AI in usability, popularity, and intelligence, consumers may misjudge a brand’s motives. They tend to interpret the brand’s use of AI as the brand’s greater focus on automation and production efficiency rather than on customer experience and product quality [12]. Consumers may believe that the enterprise chooses AI over human labor to minimize costs rather than to improve product quality. This perception reduces consumers’ perceived level of brand effort and even prompts them to question the brand’s innovative capacity and service sincerity.

Research confirms that consumers tend to perceive products requiring greater effort as having higher value [24]. The perceived design source, as an important cue, influences consumers’ inferences about the producer’s level of effort: professional designers typically invest substantial time and resources in user insights, preference research, and cultural adaptation [68], thereby being perceived as having made extraordinary efforts. In contrast, AI technology significantly improves design efficiency and reduces the time and energy the brand must invest [25]. Consumers generally recognize AI’s intelligence and efficiency and are aware that diverse designs can be generated with simple instructions. However, consumers are more willing to reward enterprises that are perceived as having invested substantial additional effort [25]. If a brand overemphasizes AI-led design, consumers may regard this as a sign of the enterprise’s unwillingness to innovate and invest, thereby reducing their purchase intention. Conversely, a high level of perceived effort significantly enhances purchase intention by improving value evaluation and trust [69]. Based on the above analysis, this study proposes the following hypothesis:

H3: Perceived brand effort plays a mediating role between the perceived design source and consumers’ purchase intention.

### 2.5. The moderating role of product type

Research confirms that consumer goods can be classified into two main categories: hedonic and utilitarian [70,71]. Hedonic products primarily satisfy consumers’ emotional desires and sensory needs, whereas utilitarian products are centered on functional value and the fulfillment of practical needs [72]. This fundamental value difference leads to a systematic divergence in consumers’ evaluations of different perceived design sources.

In the field of utilitarian products, AI-based design shows significant advantages. Since AI excels in handling tasks based on facts, logic, and functional attributes [12], it often performs better in the design of functional products. Consumers believe that AI-based design can optimize product performance and improve usage efficiency in a data-driven manner. Therefore, they exhibit higher levels of acceptance and purchase intention toward perceived AI-designed utilitarian products. In contrast, in the field of hedonic products, human designers have more advantages. Consumers generally believe that AI lacks the emotional resonance and empathy required to understand hedonic attributes [13]. As a result, they show a lower willingness to pay for hedonic products that incorporate AI [5].

On the other hand, consumers often perceive AI-designed products as lacking the necessary emotional warmth [4,18]. They view AI as automated and mechanistic, unable to adapt to and address individual uniqueness [11], and thus are skeptical of perceived AI-designed products [14]. Human designers, however, invest time and resources in understanding the target audience’s preferences, pain points, and expectations [25]. From a practical perspective, only products designed by designers who have conducted sufficient research and have a profound understanding of consumer preferences can meet the expectations of most consumers in the market. In terms of product appearance, designers must invest time and resources to ensure that the product’s visual appearance and emotional impact align with the target audience’s preferences and cultural backgrounds. Such products are perceived as conveying greater emotional warmth and value [68], thereby satisfying consumers’ emotional needs and sensory experience requirements. Based on the above analysis, this study proposes the following hypothesis:

H4: Product Type moderates the relationship between the perceived design source and consumers’ purchase intention.

**Fig 1:**
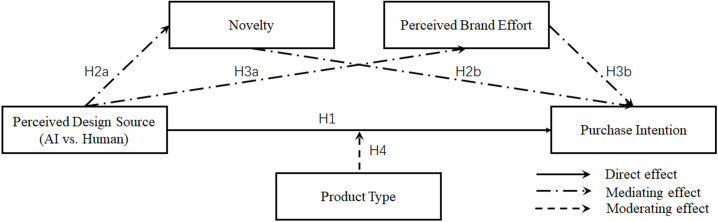
Research model is presented here for illustration.

The research model is shown in Fig 1. Building on the previous hypothesis, this study clarifies that H2 and H3 are parallel (i.e., not involving serial mediation). Additionally, in this study, product type only moderates the main effect and does not examine the moderation of the mediators’ paths.

## 3. Methods

### 3.1. Data collection

This study used questionnaires to collect data, which were divided into four parts. The first part introduced the purpose and significance of this study. Meanwhile, respondents were assured that their responses would be kept anonymous and that the data would be used solely for scientific research, not for commercial purposes. The second part of the questionnaire collected demographic information, including gender, age, educational background, shopping frequency, and occupation.

The third part of the questionnaire consists of three sequential steps. First, the experimental stimuli were carefully chosen to include 10 product images that showcase a variety of product features. Specifically, the products included a Chanel Classic Flap Bag, Dior J’adore Eau de Parfum, a limited-edition canvas art print, an Hermès Twilly silk scarf, a Baccarat Harcourt crystal vase, a Zojirushi stainless steel thermos, Nike athletic socks, a Targus backpack, a Contigo water bottle, and a Xiaomi desk lamp (pro model). These items were deliberately selected to span different product categories, as their inherent qualities could provoke distinct consumer responses. Importantly, all 10 products were real, commercially available items designed by humans. None were AI-generated designs. This approach enabled us to measure consumers’ perceptions or attributions regarding the design source (AI vs. human) based on product cues, rather than their reactions to a verifiably AI-generated stimulus. To ensure consistency and comparability of the visual materials, all images were standardized using a uniform white background, consistent lighting, and identical resolution (1200 × 800 pixels). Additionally, a pre-test with 30 participants was conducted, confirming a balanced visual appeal across all stimuli (F(9,261) = 1.23, p = 0.28). Second, after viewing these standardized product images, respondents were asked, “Do you think this product was designed by AI or a human?” based on the image they selected. Finally, following their answer to the previous question, they had to respond to, “Would you classify this product as a hedonic or a utilitarian product?” according to the chosen image.

The fourth part of the questionnaire included measurement items for core variables, such as novelty, perceived brand effort, and consumers’ purchase intention. These variables were measured using established scales from existing literature. To minimize the chance that respondents would guess answers, we randomly varied the order of these items.

The methodology complied with institutional ethical standards, having received formal approval from the Human Research Ethics Committee of the School of Art and Design at Zhengzhou University of Aeronautics (Approval Number: EA-2025054; Approval Date: 18 March 2025). In accordance with Chinese regulations, all participants were adults; no minors participated.

After obtaining ethical approval, we conducted a pre-test with 50 consumers between 2 and 15 April 2025. Results indicated that Cronbach’s alpha values for all variables exceeded 0.75 and that all items had factor loadings above 0.60. Additionally, 84% (42 individuals) found the questionnaire easy to understand, while 16% (8 individuals) experienced minor difficulties. Most participants (88%, 44 individuals) completed the questionnaire within 5–7 minutes. These findings demonstrated good reliability and validity, confirming the questionnaire’s appropriateness for full-scale data collection.

The actual data collection took place from 1 May to 28 July 2025. Offline data were gathered through intercept interviews at commercial centres across 20 Chinese cities (9 in the eastern region, 6 in the central area, and 5 in the western region). A total of 400 questionnaires were distributed, with 347 returned, yielding a response rate of 86%. 75%. All participants are adults; no minors are involved. Each participant provided written informed consent.

For the 347 returned questionnaires, invalid responses were excluded based on the following criteria: first, 15 questionnaires with response times under 3 minutes or over 9 minutes were discarded. Second, 7 questionnaires with incorrect answers to two attention-check questions were removed. Finally, logical consistency checks identified questionnaires with contradictions between purchase intention and willingness to recommend or with patterned responses, leading to the exclusion of 9 such questionnaires. Ultimately, 316 valid questionnaires remained. The descriptive statistics for these 316 valid questionnaires are presented in Table 1.

**Table 1:** Descriptive statistics (N = 316).

Demographic	Category	Frequency	Percentage	Cumulative Percentage
Gender	Male	152	48.10%	48.10%
	Female	164	51.90%	100.00%
Age	18-25 years	153	48.42%	48.42%
	26-35 years	91	28.80%	77.22%
	36-45 years	54	17.09%	94.31%
	≥45 years	18	5.69%	100.00%
Education Level	Associate degree or below	166	52.53%	52.53%
	Bachelor’s degree	123	38.92%	91.45%
	Master’s degree or above	27	8.55%	100.00%
Weekly Purchase Frequency	≤1 time	103	32.59%	32.59%
	2-4 times	150	47.47%	80.06%
	≥5 times	63	19.94%	100.00%
Occupation	Manufacturing	114	36.08%	36.08%
	Services	55	17.41%	53.49%
	Finance	36	11.39%	64.88%
	Healthcare	31	9.81%	74.69%
	Education	26	8.23%	82.92%
	Other	54	17.08%	100.00%

### 3.2. Measure

In this study, both the perceived design source and product type are categorical variables. To differentiate the categories of these variables, numerical codes are assigned to represent each category: “1” and “2” are used for the two categories of each variable, respectively. The specific approach is as follows: the questionnaire presents ten product images. Respondents are asked to select one image and then answer the question “Do you think this product was designed by AI or a human?” based on the chosen product picture. If a respondent chooses “A perceived human designed this product”, it is coded as 1; if they select “perceived AI designed this product”, it is coded as 2. After that, respondents are required to answer the question “Would you classify this product as a hedonic product or a utilitarian product?” according to the selected product image. If they classify the product as hedonic, it is coded as 1; if they classify it as utilitarian, it is coded as 2.

The measurement of novelty refers to the works of Gabriel et al. [73] and Khelladi et al. [74], which include 5 items. A representative item is “I think this product has a unique look.”

The measurement of perceived brand effort follows the approach of Li et al. [25] and comprises 3 items. A representative item is “I believe the brand has put a great deal of effort into the design of this new product.”

The purchase intention scale, adapted from Petty et al. [75], comprises 3 items. A representative item is “If conditions permit, I will purchase this product.”

For the items related to the three main variables of novelty, perceived brand effort, and purchase intention, a 7-point Likert scale was used for scoring. The scale ranges from 1 (strongly disagree) to 7 (strongly agree).

### 3.3. Tools

In this study, Mplus 8.0 was used for data analysis. The specific estimator selected was the Weighted Least Squares Mean and Variance Adjusted (WLSMV), which is particularly robust for models with categorical observed variables. This choice was based on the characteristics of our key independent variables. Specifically, both the perceived design source (AI vs. Human) and product type (Hedonic vs. Utilitarian) were measured as binary categorical variables. Unlike maximum likelihood estimation, which assumes that variables are continuous and normally distributed, the WLSMV estimator does not rely on these assumptions. It employs a weighted least-squares approach to the polychoric correlation matrix, which is specifically designed for categorical data. When applied to categorical predictors and mediators, this method can yield more accurate parameter estimates, reliable standard errors, and improved model fit indices (CFI, TLI, RMSEA, SRMR). Consequently, it ensures the statistical validity of our path analysis involving the proposed mediators (novelty, perceived brand effort) and moderator (product type).

### 3.4. Common method bias test

To address potential common method bias, both procedural and statistical measures were taken. Procedurally, respondent anonymity was ensured to mitigate concerns about evaluation. The survey included reverse-coded items and randomized item order to break response patterns and prevent guessing at possible answers. Statistically, Harman’s single-factor test was performed. The results showed that a single factor explained only 37.4% of the total variance, which is below the typical thresholds of 40% or 50%, indicating that common method bias was not a major concern in this study.

## 4. Results

### 4.1. Reliability and validity

As shown in Table 2, the constructs demonstrate strong convergent validity and reliability, with factor loadings above .60, CR values ranging from .80 to .88, and AVE values all exceeding the .50 threshold. As shown in Table 3, consumers demonstrated a significant functional orientation in their product type choices (M = 1.56, SD = 0.43). The mean value was significantly higher than the theoretical median of 1.5 (t = 2.48, p < 0.05), indicating that the proportion of respondents who selected utilitarian products was higher than those who chose hedonic products. At the cognitive level of perceived design source, the data showed that consumers were more inclined to perceived AI designed (M = 1.62, SD = 0.51). Its mean value was also significantly higher than the median (t = 4.18, p < 0.001), suggesting an increase in technological trust and a deeper cognitive penetration of AI in the design field. In addition, the square root of the AVE for each variable was greater than its correlation with any other variable, thereby supporting discriminant validity.

**Table 2:** Results of CFA and reliability and validity testing.

Dim	Item	Factor Loadings	S.E.	*p*	SMC	CR	AVE
NL	NL1	0.76	0.05	***	0.58	0.88	0.59
	NL2	0.78	0.05	***	0.61		
	NL3	0.81	0.04	***	0.66		
	NL4	0.72	0.06	***	0.52		
	NL5	0.75	0.05	***	0.56		
PBE	PBE1	0.74	0.06	***	0.55	0.80	0.57
	PBE2	0.80	0.06	***	0.64		
	PBE3	0.73	0.07	***	0.53		
PI	PI1	0.75	0.07	***	0.56	0.81	0.58
	PI2	0.82	0.06	***	0.67		
	PI3	0.71	0.07	***	0.50		

Note 1: NL = novelty; PBE = perceived brand effort; PI = purchase intention. The same below.

Note 2: ***= *p* < .001, ** = *p* <    .01, * = *p* < .05, the same below.

**Table 3:** Test of discriminant validity of variables.

Dim	Mean	SD	CR	AVE	PDS	PT	NL	PBE	PI
PDS	1.62	0.51	–	–	–				
PT	1.56	0.43	–	–	0.19	–			
NL	5.12	1.17	0.88	0.59	0.32	−0.12	**0.77**		
PBE	3.89	0.81	0.80	0.57	−0.28	0.11	0.45	**0.75**	
PI	4.56	1.05	0.81	0.58	0.15	−0.09	0.38	0.42	**0.76**

Note 1: PDS = perceived design source; PT = product type; NL = novelty; PBE = perceived brand effort; PI = purchase intention. The same below.

Note 2: Bold diagonal entries represent the square root of the AVE; the lower triangle contains the Pearson correlation coefficients.

### 4.2. Model fit

Table 4 shows the comparison of different models. The five-factor model demonstrated excellent fit across all evaluation criteria, significantly outperforming the other models. These results strongly support using the five-factor model as the best choice.

**Table 4:** Results of comparisons with alternative models.

Model	Factors	*χ²/df*	CFI	TLI	RMSEA	SRMR
Single-Factor	PDS + NL + PBE + PI + PT	6.12	0.58	0.62	0.15	0.17
Two-Factor	PDS + NL + PBE + PI, PT	5.24	0.71	0.68	0.13	0.14
Three-Factor	PDS + NL, PBE + PI, PT	3.65	0.76	0.77	0.12	0.1
Four-Factor	PDS, NL, PBE + PI, PT	3.28	0.88	0.92	0.1	0.09
Five-Factor	PDS, NL, PBE, PI, PT	2.51	0.95	0.94	0.06	0.04

### 4.3. Mediating effect

As shown in Table 5, the total effect analysis indicates that perceived AI-designed products have a significant positive effect on purchase intention (*Estimate* = 0.17, SE = 0.04, *p* < 0.001), compared with human-designed products. The 95% confidence interval [0.11, 0.23] lies entirely within the positive range, which validates the overall positive effect of perceived AI design. The direct effect is also significant (*Estimate* = 0.15, SE = 0.04, *p* < 0.001), indicating that the perceived design source has a direct effect on purchase intention, independent of the mediating variables. This may stem from consumers’ fundamental perceptions of the technological entity’s attributes, thus supporting Hypothesis 1.

**Table 5:** Test of mediating effect.

	Estimate	SE	*p*	95% CI Lower	95% CI Upper	Hypothesis
Total Effect	0.17	0.04	0.000	0.11	0.23	
Direct Effect	0.15	0.04	0.000	0.08	0.22	H1 (Y)
Indirect Effect	0.03	0.03	0.317	−0.02	0.06	
PDS → NL → PI	0.09	0.02	0.000	0.07	0.13	H2 (Y)
PDS → PBE → PI	−0.06	0.02	0.003	−0.09	−0.04	H3 (Y)

Regarding the indirect effects, the two mediating paths exhibit an antagonistic relationship. The path through which perceived AI is designed positively promotes purchase intention by enhancing product novelty (PDS → NL → PI), has an effect value of 0.09 (SE = 0.02, p < 0.001), and its 95% confidence interval [0.07, 0.13] is in the positive range. Meanwhile, the path through which perceived AI designed inhibits purchase intention by reducing perceived brand effort (PDS → PBE → PI) shows an adverse effect (*Estimate* = − 0.06, SE = 0.02, *p* = 0.003), and the 95% confidence interval [−0.09, −0.04] lies entirely within the negative range. This scenario represents a competitive mediation model, where the two mediating variables – product novelty and perceived brand effort – compete with each other to influence the relationship between the perceived design source and purchase intention. Each variable exerts its own significant but opposite-directed influence on purchase intention. The above conclusions support Hypothesis 2 and Hypothesis 3.

The total mediating effect (the algebraic sum of the two paths) is 0.03 (SE = 0.03, *p* = 0.317), with a 95% confidence interval of [−0.02, 0.06]. In the context of the competitive mediation model, the lack of significance in the total indirect effect can be understood as the competing forces of the two mediating variables essentially cancelling each other out. Although each mediating path has a significant impact on purchase intention, when their effects are combined, their opposing directions yield a non-significant net effect. This result indicates that although each path is statistically significant, their net effect is not, suggesting an asymmetric compensation mechanism between the positive cognitive path (novelty) and the negative emotional path (perceived brand effort). This dynamic balance explains the psychological contradiction in the process of technology acceptance: consumers appreciate the technological efficiency of perceived AI (an increase in novelty) while worrying about the lack of emotional warmth (a decrease in perceived brand effort), ultimately resulting in a delicate psychological equilibrium.

### 4.4. Moderating effect

Table 6 reveals the crucial moderating role of product type in the relationship between perceived design source (AI vs. human) and purchase intention, and its mechanism exhibits a significant two-way differentiation characteristic. When the product is hedonic, the promotional effect of perceived AI design on purchase intention is significantly inhibited (*Estimate* = − 0.11, *p* = 0.006), and the 95% confidence interval [−0.19, −0.03] lies entirely within the negative range. In contrast, the effect of perceived human design is strengthened (*Estimate* = 0.17, *p* < 0.001). This differentiation stems from the emotional nature of hedonic consumption: consumers regard perceived human design as conveying craftsmanship, warmth, and humanistic narratives, whereas perceived AI design is perceived as a mechanical output lacking emotional depth.

**Table 6:** Test of moderating effect.

DV	IV	Estimate	SE	*p*	95% CI Lower	95% CI Upper	Hypothesis
PI	Gender	0.05	0.03	0.096	−0.01	0.10	
	Age	−0.07	0.05	0.162	−0.17	0.03	
	Education	0.12	0.03	0.000	0.06	0.18	
	PDS	0.15	0.04	0.000	0.08	0.22	
	NL	0.25	0.05	0.000	0.16	0.34	
	PBE	−0.18	0.04	0.000	−0.26	−0.11	
	Perceived AI Design × Hedonic Product	−0.11	0.04	0.006	−0.19	−0.03	H4 (Y)
	Perceived AI Design × Utilitarian Product	0.19	0.04	0.000	0.11	0.27	
	Perceived Human Design × Hedonic Product	0.17	0.05	0.001	0.08	0.27	
	Perceived Human Design × Utilitarian Product	−0.14	0.04	0.001	−0.22	−0.07	

In the context of utilitarian products, the moderating effect shows an opposite pattern: perceived AI design significantly enhances purchase intention (*Estimate* = 0.19, *p* < 0.001), while perceived human design has an inhibitory effect (*Estimate* = −0.14, *p* < 0.001). The above conclusions validate Hypothesis 4 and are consistent with the core principle of the functional fit theory, which posits that the core value of utilitarian products lies in technological efficiency. Perceived AI-designed builds establish technological credibility through algorithm optimization, while human intervention carries the risk of human error.

## 5. Discussion

### 5.1. Interpretation of findings

This study empirically demonstrates a higher purchase intention for perceived AI-designed products than for human-designed products. This finding aligns with one branch of previous research [5,11–13]. Importantly, this alignment is understood and contextualized through a core theoretical framework——CET. Within CET, the positive net effect observed results from the specific evaluation context shaped by our research design. By including multiple product categories, our study captured a sample in which utilitarian product evaluations were prominent (as shown in the descriptive results). According to CET, this context enhances the relevance of novelty-driven positivity during the cognitive appraisal of the perceived design-source stimulus [12]. Consequently, this finding is not just a replication but a contextual validation of CET’s proposal that the appraisal outcome depends on how the stimulus interacts with the evaluative context. This explains why our results align with studies conducted in contexts favoring utilitarian or novelty-focused evaluations.

The recognition of novelty as a mediating mechanism highlights the CET-based appraisal process. Perceived AI-designed functions as a schema-incongruent stimulus [21], eliciting a positive appraisal along the novelty dimension, which in turn boosts product valuation through emotional arousal and perceived innovation [23,59]. In contrast, the parallel mediating role of perceived brand effort indicates the presence of a competing negative appraisal pathway, in which AI’s automation heuristically signals lower emotional engagement [25]. The coexistence of these opposing pathways, as anticipated by our dual-pathway CET model, directly explains the mixed findings in the literature.

The moderating effect of product type offers the key context in CET, influencing which pathway is dominant. Our results confirm that for hedonic products, which emphasize experiential goals, the perceived brand effort pathway is heightened, supporting perceived human design. For utilitarian products, which prioritize functional goals, the novelty pathway is strengthened, thereby favoring AI design [13,76]. This moderating role, demonstrated through our multi-category design, is vital to CET’s principle of context-dependence and explains the conditional effect of AI, thereby addressing prior conflicting findings across various product-focused studies.

### 5.2. Theoretical contribution

First, our study improves the methodological approach by broadening the benchmark for AI comparison. It goes beyond earlier paradigms that limited AI comparisons to professional human designers [4,5,12,13]. By using a generalized human perceived design source across a wide range of product categories in our experimental stimuli, this study offers a more ecologically valid and comprehensive comparison. This design choice enables the study’s findings to better reflect the contemporary design environment, in which AI collaborates with diverse human creators (both experts and non-experts), moving beyond the narrow “AI vs. expert” dichotomy [10].

Second, this study offers a unified theoretical framework based on CET that clarifies long-standing empirical contradictions. Incorporating CET as the primary perspective [29,77] enables us to model the two competing psychological pathways triggered by the AI-perceived design source. Our research approach, which measured both novelty [21] and perceived brand effort [24,38] simultaneously, was essential for empirically supporting this framework. This study shows that the conflicting results in previous research are not anomalies but expected outcomes of these simultaneous appraisals: a positive pathway through novelty that increases perceptions of innovation [23,35], and a negative pathway through perceived brand effort that suggests reduced authenticity [12,16]. The overall effect in any study depends on the relative strength of these pathways, transforming the understanding of the literature from scattered to systematic.

Finally, we introduce product type as a critical theoretical boundary condition and empirically demonstrate its role in determining which evaluative pathway predominates. By theoretically positioning and empirically testing product type (utilitarian vs. hedonic) as the key moderator within the CET framework, our multi-category research design was instrumental [40]. The conclusions of this study show that product type acts as the critical appraisal context that systematically directs cognitive focus: utilitarian contexts amplify the novelty pathway [12,13], while hedonic contexts amplify the perceived effort pathway [17,76]. The alignment of the main effect of this study (favoring AI) with one stream of literature is thus a specific, context-dependent instance explained by our framework, likely resulting from the evaluative context of the sample within our design. More importantly, our moderated mediation model offers a generative theoretical system [41]. It explains when (under which product contexts) and why (which cognitive pathway is amplified) future studies will align with different literature streams, thus integrating scattered findings into a coherent, predictive narrative [44].

### 5.3. Practical implications

First, companies should implement strategic task allocation based on the dual-pathway mechanism. The empirically supported dual-mediation model indicates that perceived AI, when designed together, influences purchase intention by increasing perceived novelty and reducing perceived brand effort. This calls for a sophisticated approach to managing design teams. Instead of merely restructuring organizations, companies should develop a nuanced task-allocation framework that strategically leverages the strengths of both AI and human designers. Specifically, AI’s computational power should be directed toward data-heavy tasks that maximize novelty. These include generative concept exploration, pattern recognition across consumer data, and rapid prototyping. Meanwhile, human designers should be assigned to roles that enhance perceived brand effort. They should focus on creative direction, emotional refinement of AI-generated options, storytelling, and quality control. This intentional specialization enables companies to leverage AI’s efficiency in creating novelty while preserving the human touch that signals authentic brand commitment. Implementation involves establishing clear collaboration protocols, setting evaluation metrics for both technological and human contributions, and developing training programs that foster human-AI synergy rather than treating them as separate entities.

Second, firms should adopt product-type-specific design, sourcing, and marketing strategies. The strong moderating effect of product type requires tailored approaches to these areas. For utilitarian products, where the novelty pathway is dominant, implement an AI-driven design process that emphasizes technical superiority, functional optimization, and efficiency improvements. Marketing should highlight specific technological features such as algorithmic precision, data-driven personalization, and performance enhancements. Conversely, for hedonic products where perceived brand effort is essential, adopt a human-centered approach that emphasizes craftsmanship, emotional storytelling, and artistic integrity. Marketing messages should focus on the designer’s expertise, creative process, and the unique human elements embedded in the product. Companies should establish clear guidelines to classify products along the utilitarian-hedonic spectrum and develop corresponding resource allocation systems. This approach ensures strategic alignment between perceived design source selection and the specific consumer expectations triggered by different product categories, ultimately maximizing the effectiveness of design investments.

Third, firms should adopt transparent communication that balances technical and human elements. The conflicting relationship between novelty and perceived brand effort requires carefully designed communication strategies that address both aspects at the same time. Develop a dual-focus communication framework that highlights AI’s technical sophistication while emphasizing human oversight and creative input. For perceived AI-designed products, showcase clear evidence of technological excellence, such as the size of training data, algorithmic complexity, and iterative improvement processes, to enhance the perception of novelty. At the same time, explicitly communicate the essential roles of human team members in guiding the creative vision, curating outputs, and maintaining quality standards to reduce concerns about lowered brand effort. Implement a standardized design attribution system that appears consistently across all customer touchpoints, including detailed product pages that visualize the human-AI collaboration process. This transparent communication approach helps consumers understand the complementary strengths of both perceived design sources, fostering a deeper appreciation of product value that aligns with the complex cognitive processes outlined in our research.

Finally, based on the key finding that consumers’ purchase decisions are systematically influenced by whether they believe a product is AI- or human-designed, firms should prioritize clear and accessible disclosure of the product’s perceived design source. The study’s empirical model shows that the design origin (AI vs. human) causes different consumer evaluations through novelty and perceived brand effort, with the overall effect depending on the product type. Therefore, to support accurate consumer decision-making, companies must clearly and consistently label products as “perceived AI-designed,” “perceived human-designed,” or “co-created” in all customer-facing communications, including packaging, online listings, and advertisements. For hedonic products, where perceptions of effort are more influential, such disclosures can be paired with messaging that emphasizes the human creative input or oversight involved, addressing authenticity concerns. For utilitarian products, where novelty is the main driver, communications can confidently highlight the AI’s role in promoting innovation and precision. This transparency helps consumers make informed choices, aligning their purchases with their values regarding technological innovation versus perceived human effort.

### 5.4. Limitations and future directions

First, relying on cross-sectional data is a major limitation for establishing causal claims. While the path analysis reveals associations among variables, data collected at a single point in time cannot definitively confirm the causal direction proposed by the model. Although our model suggests that perceived design source influences purchase intention through novelty and perceived brand effort, the analysis cannot completely rule out reverse causality (for example, product novelty affecting perceptions of an AI design source) or other unmeasured confounding variables. Future research should employ experimental or longitudinal designs that manipulate the design source label to observe the sequence of changes in mediators and outcomes, thereby providing stronger evidence for the causal relationship.

Second, the cultural homogeneity of our sample may limit the generalizability of our findings across diverse cultural contexts. Consumer reactions to perceived AI-designed products likely differ based on levels of technology acceptance, traditional values, and uncertainty avoidance. Future research should include cross-cultural comparisons to explore how cultural values influence technology acceptance processes, especially for perceived AI-designed products.

Third, prioritizing ecological validity in naturalistic settings came at the cost of less control over product stimuli. While this approach provides authentic consumer insights, it leaves potential confounds in product attributes insufficiently isolated. Future research should build on these findings by conducting controlled experiments with fictional brands and carefully matched products, allowing for more precise causal testing of the identified pathways.

Fourth, our sample’s age distribution warrants careful interpretation, as greater AI familiarity among younger participants may enhance observed effects via the novelty pathway. Future research should deliberately include a variety of age groups to explore potential age-related moderating effects, particularly regarding the relative influence of novelty versus perceived brand effort pathways across generations.

Fifth, our model only examined how product type moderates the direct design-source effect, leaving its potential to moderate the mediating pathways unexplored—i.e., a moderated mediation. Whether the mediation effects of novelty and perceived brand effort differ in strength between utilitarian and hedonic products remains an open question. Future research should explicitly test these conditional indirect effects to better understand the boundary conditions that shape the entire psychological process from perceived design source to purchase intention.

Sixth, using real products, especially luxury brands like Chanel and Dior, introduces a potential confounding factor: established brand equity. Consumers’ evaluations of perceived brand effort and purchase intention may be affected by their pre-existing attitudes toward these well-known brands. This reduces the ability to pinpoint the unique effect of the perceived design source. Future research should use fictional brands or carefully control for brand-specific attitudes to better distinguish the psychological impact of design source attribution from the strong connections to established brands.

## 6. Conclusions

This study empirically examines how consumers’ perceived design source (AI versus Human) affects their purchase intentions. The findings show that consumers have higher purchase intentions toward products perceived to be AI-designed, with this relationship being jointly mediated by the positive effect of novelty and the negative effect of perceived brand effort. Product type moderates the link between perceived design source and purchase intention: AI-based design is more appropriate for utilitarian products, whereas human-led design is better suited to hedonic products. These results not only advance marketing theory but also provide a theoretical foundation for companies to develop differentiated design strategies.
